# HERC1 deficiency causes osteopenia through transcriptional program dysregulation during bone remodeling

**DOI:** 10.1038/s41419-023-05549-x

**Published:** 2023-01-12

**Authors:** Leonardo Pedrazza, Arturo Martinez-Martinez, Cristina Sánchez-de-Diego, José Antonio Valer, Carolina Pimenta-Lopes, Joan Sala-Gaston, Michal Szpak, Chris Tyler-Smith, Francesc Ventura, Jose Luis Rosa

**Affiliations:** 1 Departament de Ciències Fisiològiques, Universitat de Barcelona, IDIBELL, L’Hospitalet de Llobregat, Spain; 2 Wellcome Sanger Institute, Wellcome Genome Campus, Hinxton, UK

**Keywords:** Extracellular signalling molecules, Transcription

## Abstract

Bone remodeling is a continuous process between bone-forming osteoblasts and bone-resorbing osteoclasts, with any imbalance resulting in metabolic bone disease, including osteopenia. The *HERC1* gene encodes an E3 ubiquitin ligase that affects cellular processes by regulating the ubiquitination of target proteins, such as C-RAF. Of interest, an association exists between biallelic pathogenic sequence variants in the *HERC1* gene and the neurodevelopmental disorder MDFPMR syndrome (macrocephaly, dysmorphic facies, and psychomotor retardation). Most pathogenic variants cause loss of HERC1 function, and the affected individuals present with features related to altered bone homeostasis. *Herc1*-knockout mice offer an excellent model in which to study the role of HERC1 in bone remodeling and to understand its role in disease. In this study, we show that HERC1 regulates osteoblastogenesis and osteoclastogenesis, proving that its depletion increases gene expression of osteoblastic makers during the osteogenic differentiation of mesenchymal stem cells. During this process, HERC1 deficiency increases the levels of C-RAF and of phosphorylated ERK and p38. The *Herc1*-knockout adult mice developed imbalanced bone homeostasis that presented as osteopenia in both sexes of the adult mice. By contrast, only young female knockout mice had osteopenia and increased number of osteoclasts, with the changes associated with reductions in testosterone and dihydrotestosterone levels. Finally, osteocytes isolated from knockout mice showed a higher expression of osteocytic genes and an increase in the *Rankl/Opg* ratio, indicating a relevant cell-autonomous role of HERC1 when regulating the transcriptional program of bone formation. Overall, these findings present HERC1 as a modulator of bone homeostasis and highlight potential therapeutic targets for individuals affected by pathological *HERC1* variants.

## Introduction

Bone is a highly dynamic tissue that is subject to continuous remodeling throughout an individual’s life. The bone remodeling process maintains skeletal homeostasis in adults through a process in which new tissue from mesenchymal-derived osteoblasts replaces damaged or old bone degraded by hematopoietic-derived osteoclasts. Under physiological conditions, osteoblasts equivalently replace and deposit the same amount of bone reabsorbed by osteoclasts to maintain homeostasis, and imbalances between resorption and bone formation can lead to pathology [1–3].

Ubiquitin regulates key proteins related to bone homeostasis. Protein ubiquitination (or ubiquitylation) is a three-step process that involves activating ubiquitin by ATP-dependent activating enzyme E1, transferring ubiquitin from E1 to the conjugating enzyme E2, and transferring ubiquitin from E2 to the target protein mediated by the ubiquitin ligase enzyme E3. This ubiquitin ligase binds specifically to the target protein and transfers ubiquitin to a lysine residue or to the N-terminal amino acid residue. Through this process, ubiquitin tags proteins and causes structural modifications such as monoubiquitination, multiubiquitination, or polyubiquitination. These post-translational modifications are important for protein function and can affect the localization, activity, and degradation of proteins [4, 5].

Several bone remodeling studies have shown that ubiquitin ligases may play a key role in regulating bone metabolism [6, 7]. HERC proteins are ubiquitin ligases of the Homologous to E6AP Carboxyl-Terminus (HECT) family and contain an additional Regulator of Chromosome Condensation 1 (RCC1)-like domain as a structural feature [8]. Of the six *HERC* genes described in humans, two (*HERC1* and *HERC2*) encode large HERC proteins with molecular weights above 520 kDa. Of these, *HERC1* somatic mutations have been identified in cancers such as leukemia, breast cancer, and non-melanoma skin cancer [9, 10] and germ-line mutations have been reported in neuronal disorders [11, 12]. In this context, genetic studies have related *HERC1* mutations with autism spectrum disorders, considering it a predictor of autism risk [13, 14]. The common features observed in individuals with homozygous or compound heterozygous mutations in the *HERC1* gene led to the identification of the autosomal recessive neurodevelopmental disorder MDFPMR syndrome (macrocephaly, dysmorphic facies, and psychomotor retardation) (OMIM # 617011) [15–19]. Altered bone homeostasis could account for several features observed in these individuals, such as macrocephaly, dysmorphic facies, prominent forehead, long fingers, and vertebral column abnormalities [11]. Most of the *HERC1* mutations associated with MDFPMR syndrome are frameshift mutations that cause truncated HERC1 proteins and a loss of function [15–18].

Despite the significant advances in our understanding of the roles of ubiquitin ligases in mediating bone remodeling, no study to date has investigated the role of HERC1. Among other factors, the lack of suitable animal models for the analysis of HERC1 physiological function has limited these studies. In this analysis, we used a *Herc1*-KO mouse model to address the role of HERC1 in bone homeostasis.

## Materials and methods

### Cell cultures

Mesenchymal stem cells (MSCs) were isolated from bone marrow as previously reported [20, 21]. Primary osteocytes were isolated as previously described [22].

### Osteoblast differentiation

When MSCs reached a confluence of 90%–100%, the culture medium (DMEM with 20% FBS, 2 mM l-Glutamine, and 100 U/mL penicillin/streptomycin) was replaced with a differentiation medium as previously described [23]. It was composed of α-MEM (Biological Industries) supplemented with 10% FBS, 2 mM L-Glutamine, 1 mM sodium pyruvate, 50 µM ascorbic acid, 10 mM β-glycerol phosphate, and 100 U/mL penicillin/streptomycin. This medium was renewed every two days until 21 days of differentiation.

### Alizarin Red staining and alkaline phosphatase activity

Cells were fixed in 4% paraformaldehyde for 15 minutes at room temperature. Alizarin Red staining (ARS Staining Quantification Assay, ScienCell #8678) and alkaline phosphatase activity measurement (Sigma-Aldrich #SCR004) were performed according to the manufacturer’s instructions.

### Lentiviral particle production and target cell infection

Lentiviral vectors were produced in HEK 293 T cells. Cells were transfected with pMD2.G, psPAX2 (VSV-G), and either empty pLKO.1-Puro or pLKO.1‐shHERC1, using the calcium phosphate method. Media containing lentiviral particles were collected, filtered (Millipore SLHV033RB), and stored in aliquots at −80 °C. Target cells were seeded at a confluence of 50–60% in a six-well plate before adding 300 μL of the medium containing the lentiviral vectors to each well. Fresh medium, supplemented with 5 μg/mL polybrene, was added to a total volume of 1 mL. Media with lentiviral vectors were removed the next day, and 5 μg/mL puromycin was added for selection after 24 h. A MISSION shRNA clone of mouse HERC1 (TRCN0000087441) was purchased from Sigma-Aldrich. Dr. David Root gifted the plasmid vector pLKO.1–TRC control (Addgene plasmid #10879) [24] and Dr. Didier Trono gifted the VSV-G envelope expressing plasmid pMD2.G (Addgene plasmid #12259) and the lentivirus packaging plasmid psPAX2 (Addgene plasmid #12260).

### Immunoblotting

Lysates were prepared with an NP40 buffer, as previously described [25]. We used a Tris-Acetate PAGE system to analyze the samples [26], with a gel documentation system (LAS-3000 Fujifilm). Antibodies used: anti-CHC (Santa Cruz Biotechnology #sc12734), anti-C-RAF (BD Biosciences #610151), anti-p-ERK1/2 (Sigma-Aldrich #M8159), anti-ERK1/2 (Cell Signaling #9102), anti-p-p38 (Cell Signaling #9211), anti-p38 (Santa Cruz Biotechnology #sc-535), anti-Vinculin (Santa Cruz Biotechnology #sc-25336), and anti-HERC1 (410) [27].

### Animal model

The Wellcome Trust Sanger Institute generated the *Herc1*-KO mouse strain (C57BL/6N-*Herc1*^*em3(IMPC)Wtsi*^*/Wtsi*) used in this study [28–31]. A Crispr/Cas9-mediated deletion resulted in a 191 bp deletion that included exon 8 of the *Herc1* gene located in chromosome 9. Animals had *ad libitum* access to food and water in an environment with a 12-h light/dark cycle set at 22–24 °C. The sample size was estimated from our previous publications. All mice were age (8- or 32-week-old) and sex-matched and then randomized for experiments. No animal was excluded from the analysis. Mice were euthanized and samples were collected for histological, micro-CT, gene expression, and hormonal analysis. Blinding was not done. All animal protocols were approved by the Ethics Committee for Animal Experimentation of the University of Barcelona and the Generalitat of Catalunya (Spain).

### Histological analysis

Femurs were fixed in 4% paraformaldehyde for 24 h at 4 °C, decalcified in EDTA solution (14%, pH 7.4), and embedded in paraffin after 6 weeks. Samples were cut into 7-μm sections and stained with hematoxylin/eosin (H&E), tartrate resistant acid phosphatase (TRAP), or Masson’s trichrome. Osteoclasts numbers were measured in isolated trabeculae from TRAP images. For each trabecula, the number of osteoclasts on its surface was divided by its perimeter. At least 10 trabeculae from each animal were used. H&E images were also used to count osteoblasts on the surface of trabeculae and to detect osteocytes inside the cortical bone, reported as the number divided by the bone area analyzed).

### Gene expression

For gene expression analysis in femurs, soft tissue was removed by a scalpel. Then, the femur end was cut and the bone marrow was eliminated by placing the femur in a perforated 0.6 mL centrifuge tube inserted into a 1.5 mL tube followed by centrifugation for 30 s at 5700 × *g*. This isolated bone fraction was cut into small pieces and homogenized in TRIsure reagent (Bioline, London, UK) with a Polytron PT 2100 at 26000 rpm for 45 s. RNA isolation was performed using the standard TRIsure protocol. After RNA isolation, it was reverse-transcribed using a High-Capacity cDNA Reverse Transcription Kit (Applied Biosystems). Quantitative PCR was performed with an ABI Prism 7900 HT Fast Real-Time PCR System and a Taqman 5’-nuclease probe method (Applied Biosystems) with a SensiFAST Probe Hi-ROX Mix (Bioline). PCR data acquisition and analysis used the Sequence Detector software (Applied Biosystems, SDS version 2.3). All transcripts were normalized to *Gapdh* or TATA binding protein (*Tbp*) expression.

### Hormonal analysis

Serum samples from 8-week-old mice were analyzed with a testosterone ELISA kit (ADI-900-065, Enzo Life Sciences), a 17β-Estradiol ELISA kit (ADI-900-174, Enzo Life Sciences), and a dihydrotestosterone ELISA kit (#KA1886, Abnova), according to the manufacturer’s instructions.

### Micro-CT analysis

Animals were sacrificed and their femurs were dissected and fixed in 4% paraformaldehyde for 24 h. According to recommendations from the American Society of Bone and Mineral Research, we acquired high-resolution images from the femur using a micro-computed tomography (micro-CT) imaging system (Skyscan 1272, Bruker microCT, Kontich), as previously described [22].

### Statistical analysis

Data were analyzed using GraphPad Prism 8 software and expressed as means ± standard error of the mean (SEM) for *n* independent experiments, as indicated in the figure legends. Differences between groups were analyzed using the Student’s *t* test or one-way analysis of variance with Bonferroni’s multiple comparison test. Differences were considered significant at *p < 0.05, **p < 0.01, and ***p < 0.001.

## Results

### HERC1 regulates osteoblastic differentiation

**Fig. 1:**
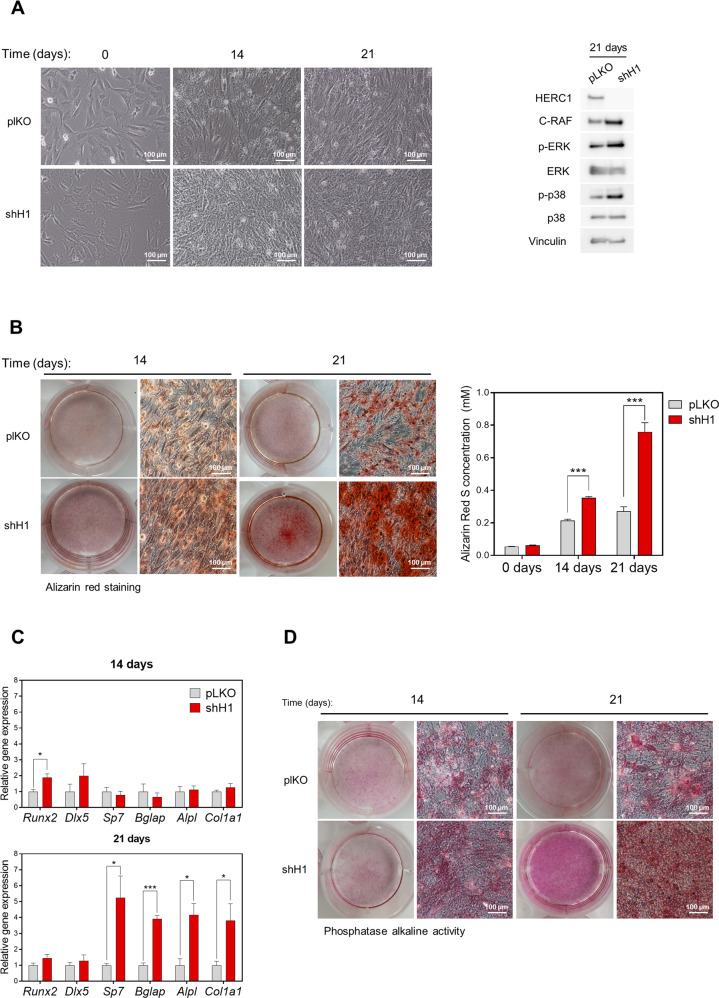
HERC1 regulates the osteoblastic differentiation. Bone marrow derived MSCs were infected with lentiviral particles carrying either the empty plKO vector as a negative control (plKO) or an shRNA against HERC1 (shH1) and were further treated with an osteoblastic differentiation medium. **A** Representative images of differentiation process at 0, 14, and 21 days. Lysates were analyzed by immunoblotting, using specific antibodies against the indicated proteins. **B** Alizarin Red staining. Representative images were acquired by optical microscopy at the times indicated. Quantification is shown (*n* = 5). **C** Osteoblastic gene expression of MSCs undergoing differentiation at the times indicated. The mRNA expression levels were measured by RT-qPCR and normalized to *Tbp* expression (*n* = 4). **D** Cells were fixed and stained for alkaline phosphatase activity. Representative images are shown. Data are expressed as mean ± SEM. Significant differences: *p < 0.05; **p < 0.01; ***p < 0.001.

Confluent bone marrow derived MSCs differentiated into osteoblasts after approximately 3–4 weeks in culture with a specific differentiation medium. We used this cell model of osteoblastogenesis [32] to assess whether HERC1 could regulate osteoblastic differentiation. MSCs were infected with mock lentivirus (plKO) or with lentivirus expressing shRNA against HERC1 (shH1) and their differentiation studied for 3 weeks (Fig. 1A). The downregulation of HERC1 protein expression by shH1 was maintained over the time studied (Fig. 1A, right). Under these conditions, HERC1 depletion increased protein levels of C-RAF and phosphorylated ERK and p38 (Fig. 1A, right). Alizarin Red staining, used to evaluate calcium deposits during osteoblastic differentiation, increased in control cells (Fig. 1B) and increased to a greater degree in HERC1 knockdown cells (Fig. 1B), consistent with the differences in cell morphology detected by light microscopy (Fig. 1A). We assessed the expression of osteoblast-specific genes by RT-qPCR to confirm the stimulation of osteoblastic differentiation by HERC1 depletion, revealing a significant increase in mRNA levels of early markers, such as transcription factor *Runx2*, in HERC1-depleted cells compared to control cells at 14 days (Fig. 1C). Furthermore, mRNA levels of later markers of osteoblastic differentiation, such as *Sp7* (osterix)*, Bglap* (osteocalcin)*, Alpl* (alkaline phosphatase), *and Col1a1* (collagen), were also significantly increased in HERC1-depleted cells compared to control cells at 21 days (Fig. 1C). Consistent with *Alpl* mRNA levels, alkaline phosphatase activity was greater in HERC1-depleted cells at 21 days (Fig. 1D). Altogether, these results confirmed the involvement of HERC1 in regulating osteoblastic differentiation.

### Female-associated osteopenia in young *Herc1*-KO mice

**Fig. 2:**
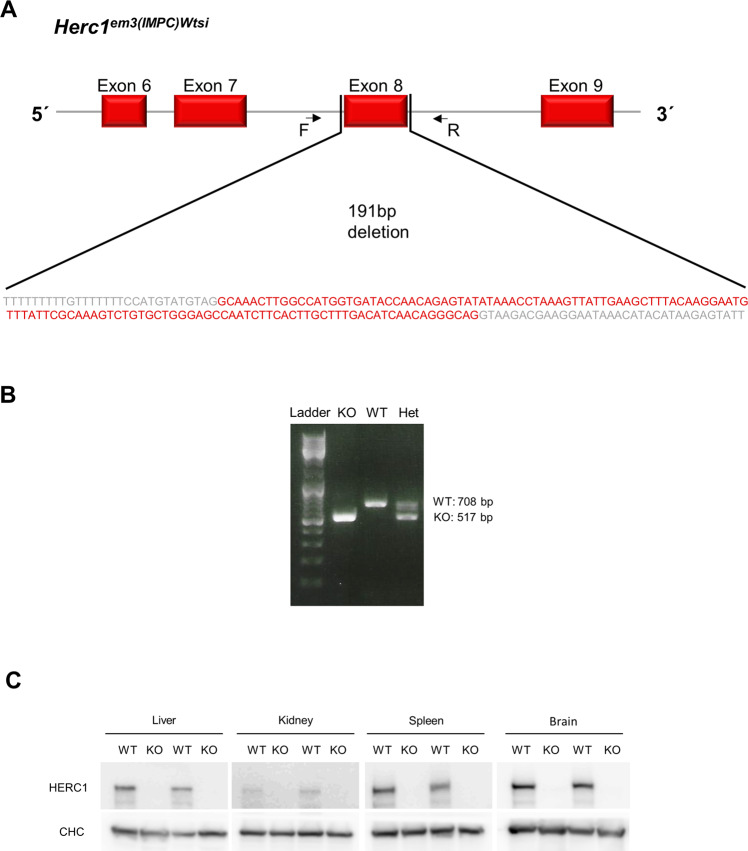
*Herc1*-knockout mouse. **A** A mouse C57BL/6N-*Herc1*^*em3(IMPC)Wtsi*^*/Wtsi* model was generated at the Wellcome Trust Sanger Institute. A Crispr/Cas9 mediated deletion resulted in a 191-bp deletion that included exon 8 on the *Herc1* gene. **B** Genotyping. Representative PCR analysis with the Forward (F) and Reverse (R) primers shown. The Thermo Scientific (#SM0331) DNA Ladder mix was used as a molecular weight marker. **C** Lysates from the indicated tissues were analyzed by immunoblotting using specific antibodies against the indicated proteins. Clathrin heavy chain (CHC) was used as the control protein. Het heterozygote, KO knockout, WT wild-type.

Studies with in vivo models are necessary to analyze the physiological role of HERC1 in bone homeostasis. Thus, we used a *Herc1*-KO mouse strain (C57BL/6N-*Herc1*^*em3(IMPC)Wtsi*^*/Wtsi*) generated by a Crispr/Cas9-mediated deletion that included exon 8 on the *Herc1* gene (Fig. 2A). Genotyping by PCR revealed a lower band due to the 191-bp deletion in the KO alleles (Fig. 2A, B). Immunoblotting confirmed the lack of HERC1 protein expression in all analyzed tissues (Fig. 2C).

**Fig. 3:**
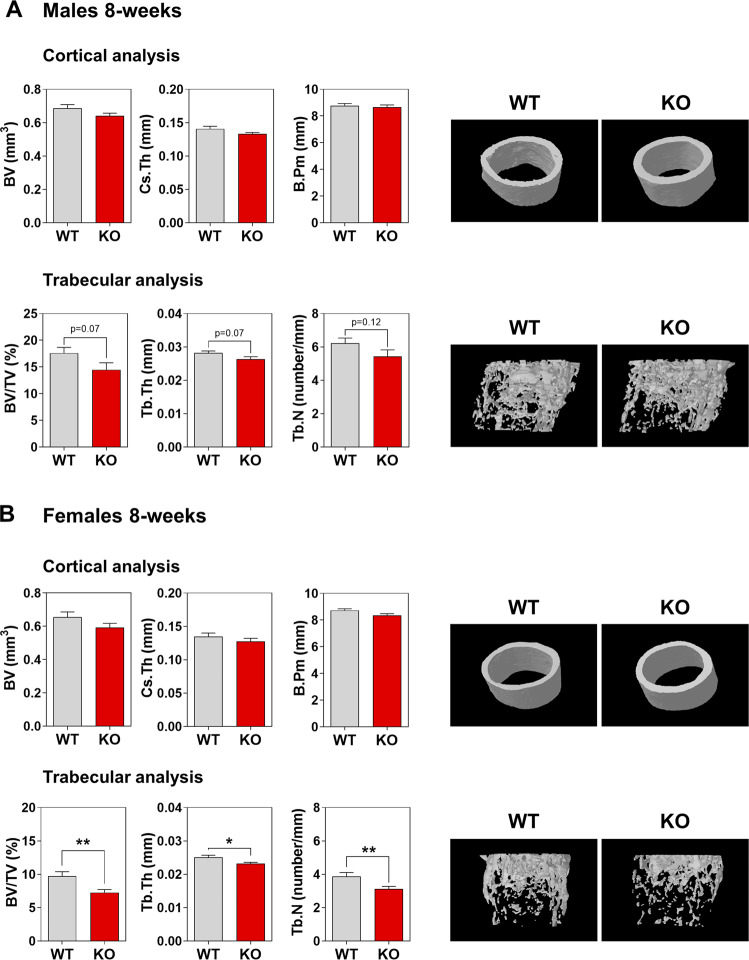
Female-associated osteopenia in young *Herc1*-knockout mice. Bone composition by micro-CT analysis of 8-week-old mice. Cortical and trabecular analysis of distal femurs from male (**A**) and female (**B**) mice. Representative images are shown. Data are expressed as mean ± SEM (*n* = 8–12). Significant differences are relative to WT mice of the same sex. *p < 0.05; **p < 0.01. BV bone volume, B.Pm bone perimeter, BV/TV bone volume over total volume, Cs.Th cortical thickness, Tb.N trabecular number, Tb.Th trabecular thickness, KO knockout, WT wild-type.

We then assessed bone composition by micro-CT scan imaging of the distal femurs of 8-week-old mice. Cortical analysis of knockout (KO) male mice demonstrated no significant modification compared to male wild-type (WT) mice (Fig. 3A). Although not significant, we observed a trend toward lower values in the trabecular analysis of KO male mice (Fig. 3A). In females, both the WT and KO mice had normal cortical results (Fig. 3B), but surprisingly, significant alterations were present between WT and KO mice in the trabecular region (Fig. 3B). The distal femurs presented less trabecular bone volume (bone volume over total volume) due to the significantly lower trabecular number (Tb.N) and thickness (Tb.Th) (Fig. 3B). These data show an unexpected osteopenia in young KO female mice.

### HERC1 deficiency causes an imbalance in bone remodeling

**Fig. 4:**
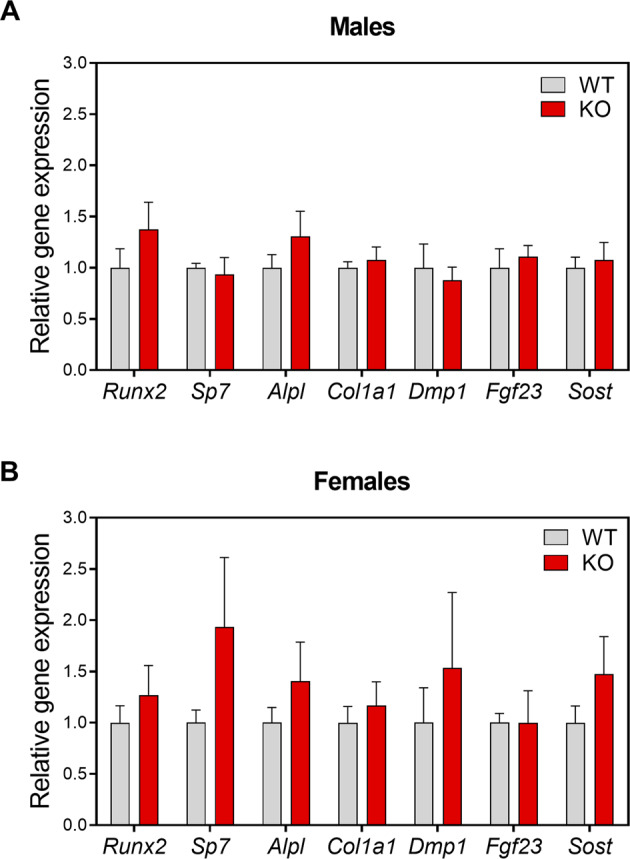
Expression levels of osteoblastic genes in *Herc1* knockout mice. **A**, **B** We analyzed mRNA isolated from the femurs of 8-week-old WT or *Herc1*-KO mice by RT-qPCR, normalized to *Gapdh* expression (*n* = 4–9) and expressed as mean ± SEM. KO knockout, WT wild-type.

**Fig. 5:**
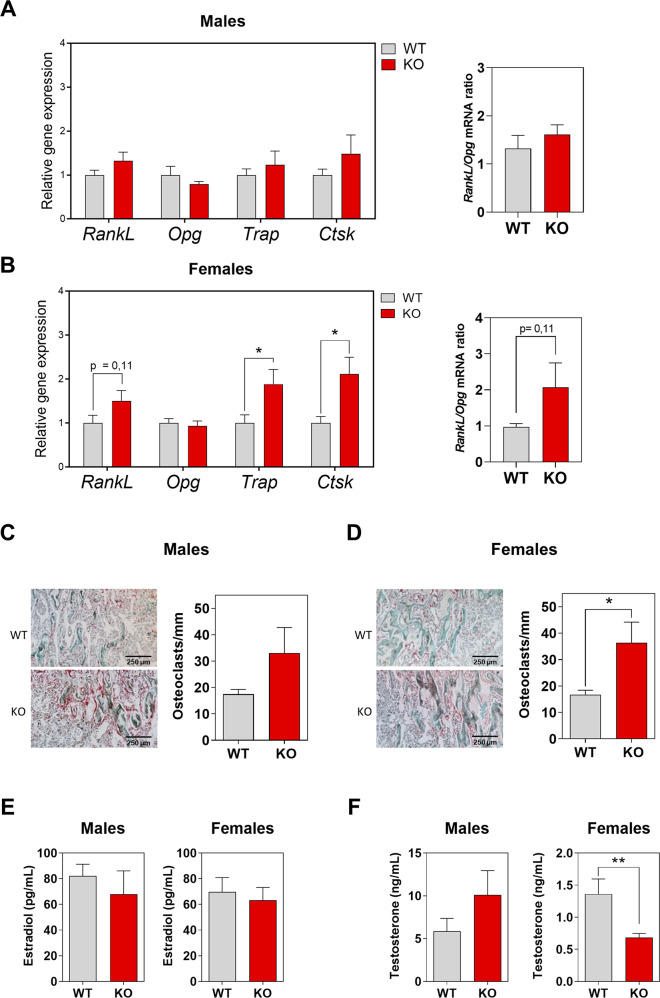
HERC1 induces osteoclastogenesis. **A**, **B** We analyzed mRNA isolated from the femurs of 8-week-old WT or *Herc1* KO mice by RT-qPCR, normalized to *Gapdh*, expression and show the *Rankl/Opg* mRNA ratio (*n* = 5–9). **C** Histological analysis of femurs obtained from mice. **C**, **D** Representative images of longitudinal sections of femur from male and female KO mice stained with TRAP. Images were taken at ×4 magnification. Osteoclasts number per surface of trabecular bone in both sexes in WT and *Herc1*-KO mice (*n* = 3–5). Estradiol (**E**) and testosterone (**F**) levels in the serum of WT and *Herc1*-KO mice were measured by ELISA (*n* = 10–15). Data are expressed as mean ± SEM. Significant differences are relative to WT mice. *p < 0.05; **p < 0.01. KO knockout, WT wild-type.

The osteopenia observed in 8-week-old female KO mice could suggest an imbalance between bone-forming osteoblasts and bone-resorbing osteoclasts. To check this possibility, we analyzed the mRNA expression of osteoblast and osteocyte genes, starting with osteoblastogenesis. The bones from WT and KO male mice had comparable expression of osteoblastogenic genes (Fig. 4A). However, female KO mice showed a tendency to have an increased expression of osteoblastogenic genes (Fig. 4B). To focus on osteoclastogenesis, we looked at the receptor activator of nuclear factor κB ligand (RANKL) and osteoprotegerin (OPG), a decoy receptor for RANKL. Although both are secreted by osteoblasts and osteocytes, osteocytes are the most abundant (>90%) bone cells. RANKL and OPG act as positive and negative regulators of osteoclastogenesis, respectively. The regulation of osteoclast differentiation, activation, and survival depends on the RANKL/OPG ratio, which affects the balance between bone formation and resorption [33–35]. Thus, we analyzed *Rankl* and *Opg* mRNA levels from long bones of 8-week-old mice. Although male KO mice did not show differences in *Rankl* and *Opg* mRNA levels compared with male WT mice (Fig. 5A), female KO mice showed increased *Rankl* mRNA expression compared with female WT mice that caused an increase in the *Rankl/Opg* ratio (Fig. 5B). These increases suggest an augmentation of osteoclastogenesis in female KO mice. To clarify this point, we also analyzed the expression of genes encoding osteoclast-specific proteins, such as tartrate resistant acid phosphatase type 5 (*Trap*) and Cathepsin K (*Ctsk*). While male KO and WT mice did not show differences in *Trap* and *Ctsk* mRNA levels, female KO mice showed higher levels of these mRNAs compared with female WT mice (Fig. 5A, B). We then performed histomorphometric measurements of TRAP-stained femoral sections to confirm that the increased osteoclastogenesis in female KO mice resulted from increases in the *Rankl/Opg* ratio and the *Trap* and *Ctsk* mRNA levels. This revealed a higher number of osteoclasts on the surface of trabeculae from female KO mice (Fig. 5C–D), but without changes in the numbers of osteoblasts and osteocytes (Supplementary Fig. 1).

Next, we investigated whether changes in sex hormone levels could have led to the osteopenia in 8-week-old KO female mice. Although this revealed no significant differences in serum estradiol concentrations between KO and WT mice in either sex (Fig. 5E), or in testosterone concentrations between male KO and WT mice (Fig. 5F), we observed a significant reduction in the testosterone concentration for female KO mice compared with their WT controls (Fig. 5F). Analysis of the dihydrotestosterone concentration revealed its reduction exclusively in female KO mice (Supplementary Fig. 2). Taken together, these results show a correlation between female-associated osteopenia, with lower concentrations of testosterone and dihydrotestosterone, in young *Herc1*-KO mice.

### HERC1 deficiency causes osteopenia in adult mice

**Fig. 6:**
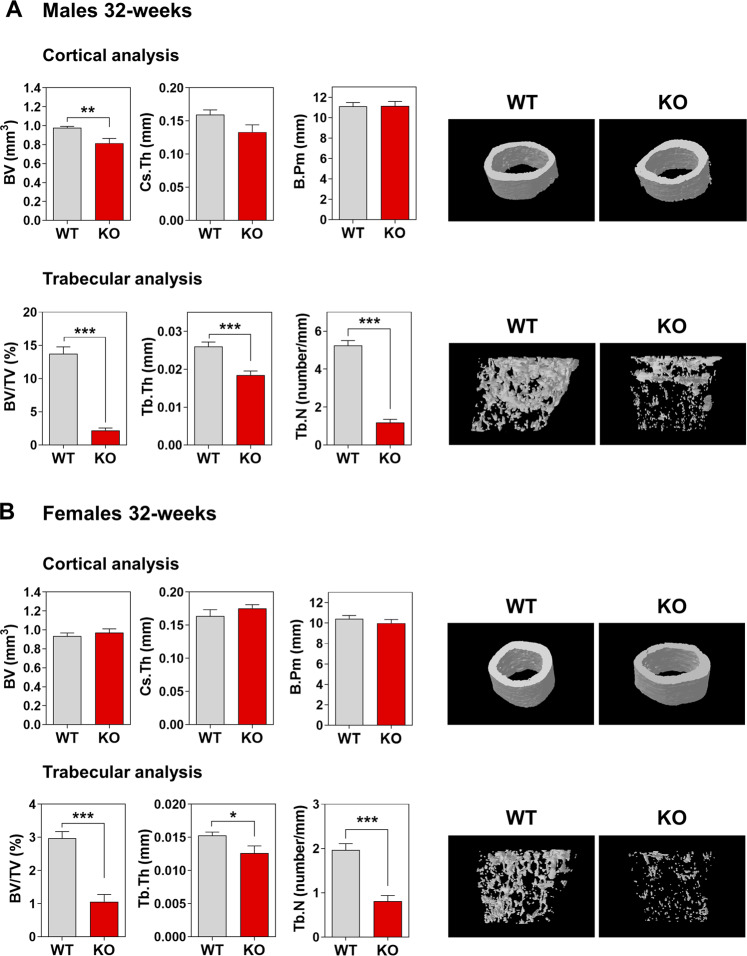
HERC1 deficiency causes osteopenia in adult mice. Bone composition by micro-CT analysis of 32-week-old mice. Cortical and trabecular analysis of distal femurs from male (**A**) and female (**B**) mice. Representative images are shown. Data (*n* = 6–12) are expressed as mean ± SEM. Significant differences are relative to WT of the same sex. *p < 0.05; **p < 0.01; ***p < 0.001. BV bone volume, B.Pm bone perimeter, BV/TV bone volume over total volume, Cs.Th cortical thickness, Tb.N trabecular number, Tb.Th trabecular thickness, KO knockout, WT wild-type.

Although we observed osteopenia in female KO mice, we only observed a trend to lower values in the trabecular analysis of male KO mice (Fig. 3A). This led us to consider how osteopenia evolves in adult female KO mice and whether male KO mice develop osteopenia as adults. Thus, we completed a bone composition study in 32-week-old mice. Cortical analysis at this age showed no differences between KO and WT female mice (Fig. 6), but conversely, trabecular analysis of distal femurs revealed a strong decrease in the morphometric values of female KO mice compared to WT controls (Fig. 6B). These data confirm the osteopenia observed in young female mice and an increase in its severity in adult females. Interestingly, cortical and trabecular analysis in adult male mice showed strong decreases in the morphometric values, indicating severe osteopenia in adult male KO mice as well (Fig. 6A).

### HERC1 regulates gene expression in osteocytes

**Fig. 7:**
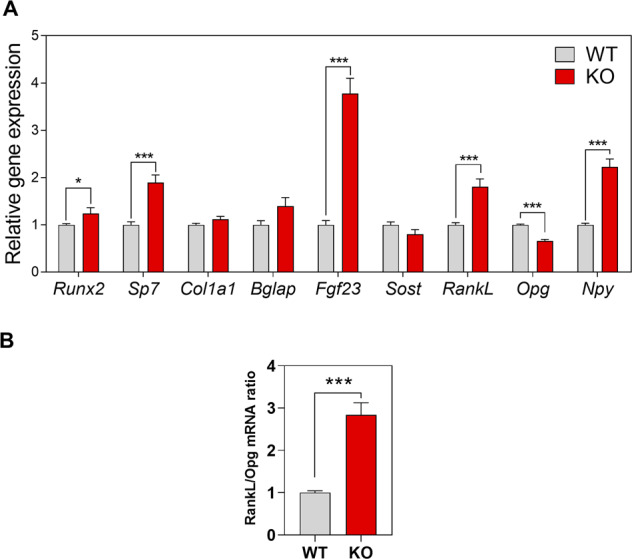
HERC1 regulates gene expression in osteocytes. **A** Osteocytes were isolated from bones of WT or *Herc1*-KO mice. Cells were lysed and mRNA analyzed by RT-qPCR. Osteocyte gene expression was normalized to *Tbp* expression. **B** The *Rankl/Opg* mRNA ratio. Data are expressed as mean ± SEM. Significant differences are relative to WT mice. *p < 0.05; ***p < 0.001. KO knockout, WT wild-type.

Paracrine hormone regulation is an important factor controlling bone homeostasis [36, 37]. Nevertheless, we cannot exclude a role for HERC1 in hormone-independent bone homeostasis. To identify cell-autonomous effects, we analyzed primary osteocytes from KO animals. Osteocytes are the major source of RANKL for osteoclast formation and bone remodeling [33–35]. We therefore hypothesized that the observed increase in *Rankl* mRNA and *Rankl/Opg* ratio (Fig. 5B) would be much more evident in RANKL-producing principal cells independent of paracrine signaling. We observed a significant difference in *Rankl* and *Opg* mRNA expression, resulting in a large increase in the *Rankl/Opg* ratio in osteocytes from KO mice compared with WT animals (Fig. 7). Osteocytes from KO mice also showed increased expression of other osteocyte genes, such as *Runx2, Sp7, Fgf23* (fibroblast growth factor 23), and *Npy* (neuropeptide-Y) (Fig. 7). These results agree with those from the in vivo analysis of osteoclastogenic markers in KO mice (Fig. 5) and the observations during the osteoblast differentiation of MSCs (Fig. 1). In conclusion, these data show a relevant cell-autonomous role of HERC1 in regulating the transcriptional program in osteocytes.

## Discussion

**Fig. 8:**
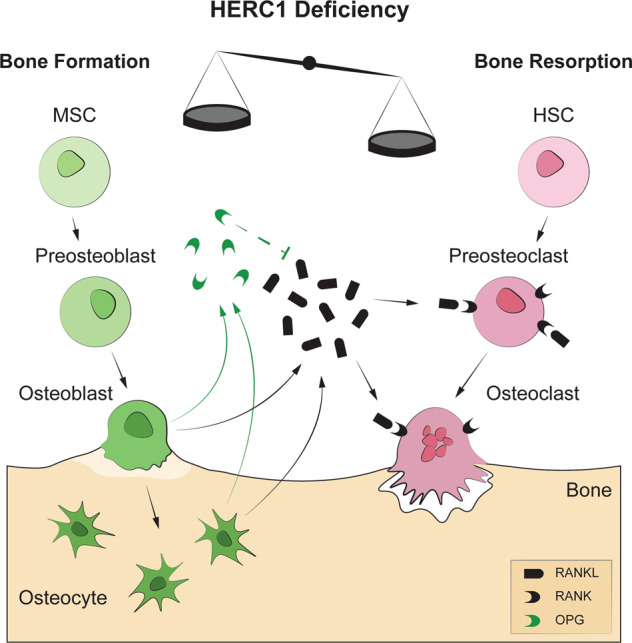
Schematic model showing the role of HERC1 in bone homeostasis. Osteoblasts and osteocytes secrete RANKL, which then binds to its receptor (RANK) on the membranes of pre-osteoclasts/osteoclasts to increase osteoclastic differentiation and activation, resulting in bone resorption. Secreted OPG then binds to RANKL, inhibiting RANK signaling and bone resorption. Morphometric analysis of bones from adult *Herc1*-KO mice revealed osteopenia, indicating an imbalance between bone formation and bone resorption. This led to increases in the *Rankl/Opg* ratio and in the number of osteoclasts. HSC hematopoietic stem cell, MSC mesenchymal stem cells, OPG osteoprotegerin, RANKL RANK ligand, RANK receptor activator of nuclear factor κB.

This study provides the first evidence that HERC1 controls bone homeostasis and that loss of its function causes osteopenia. Our data show that HERC1 depletion in MSCs increases their differentiation to osteoblasts and raises both C-RAF levels and the phosphorylation of ERK and p38, enhancing the gene expression of key transcription factors for osteoblast differentiation. Analyzing the osteocytes isolated from *Herc1*-KO mice confirmed the increases in *Runx2* and *Sp7* gene expression following *Herc1* deletion. Despite the increased gene expression of osteoblastic differentiation activators, morphometric analysis of bones from adult *Herc1*-KO mice showed osteopenia, indicating an imbalance between bone formation and bone resorption. Analysis of the expression of genes that regulate osteoclast formation and activation revealed an increase in the *Rankl/Opg* ratio, suggesting an increase in osteoclastic activity. Subsequent histological analysis confirmed the increased number of osteoclasts. These data indicate that HERC1 regulates osteoblastogenesis and osteoclastogenesis for bone homeostasis, and that HERC1 deficiency activates these cellular processes by increasing the RANKL/OPG ratio to increase the number of osteoclasts and cause an imbalance in bone remodeling (Fig. 8). This RANKL/OPG-dependent mechanism is clearly observed in 8-week-old mice. In 32-week-old mice, although they have more advanced osteopenia, no significant differences in the RANKL/OPG ratio are observed between KO and WT mice (not shown). These data suggest that in 32-week-old adult mice, the cumulative effect of bone resorption is observed throughout this period of life, although we cannot rule out that additional mechanisms independent of RANKL are acting at older ages.

Mitogen-activated protein kinases (MAPKs), such as ERK and p38, are key players in skeletal development and bone homeostasis [38–40]. In vitro and genetic studies have shown the positive effect of ERK activation on osteoblast differentiation [41–46], which phosphorylates RUNX2 and increases its transcriptional activity [47]. In osteoblasts, ERK activation also increases RANKL expression and regulates RANKL-induced osteoclastogenesis [45]. Comparable research has shown the need of p38 activation during osteoblast differentiation and osteoclastogenesis [38, 40, 41, 48–50], with p38-mediated phosphorylation of osteoblast transcription factors enhancing their transcriptional activity and promoting osteogenic progression [38, 39]. In this context, RANKL acts as an osteoclastogenic factor binding to its receptor (RANK) on the plasma membrane of osteoclasts and inducing ERK and p38 activation, thereby promoting osteoclast differentiation, migration, and bone resorption [40].

MAPKs form signaling cascades where upstream kinases phosphorylate and activate downstream kinases. RAF proteins are MAPK kinase kinases that phosphorylate and activate MEK proteins, which in turn, phosphorylate and activate ERK proteins. These MAPKs constitute the RAF/MAPK/ERK signaling cascade involved in regulating a wide variety of cellular processes, including proliferation and differentiation [51]. C-RAF, an isoform of RAF, has been specifically identified as a substrate for the ubiquitin ligase HERC1 [25]. Ubiquitination of C-RAF targets this kinase for proteasomal degradation, and HERC1 deficiency stabilizes C-RAF, with the resulting increase in its protein levels sufficient to activate ERK. Moreover, HERC1 deficiency activates p38 dependent on RAF activity [52]. Now, our data show that HERC1 depletion during osteogenic differentiation causes an increase in C-RAF levels, thereby activating ERK and p38 (Fig. 1). More importantly, these observations suggest a molecular mechanism for the role of HERC1 in regulating bone homeostasis. The increased ERK and p38 activity caused by C-RAF stabilization following HERC1 deficiency appears sufficient to activate the osteoblastic transcriptional program (Fig. 1). This activation, in turn, would increase mRNA levels of *Rankl* and decrease mRNA levels of *Opg* in osteocytes (Fig. 7). The expression of these factors would then increase the RANKL/OPG ratio, activating RANKL-induced osteoclastogenesis to produce an imbalance in bone homeostasis, and ultimately causing osteopenia. (Fig. 8). Since ERK and p38 activation is also necessary for osteoclastogenesis [40], we cannot discard an additional role of HERC1 on osteoclasts. Comparative genetic studies, likely largely using conditional KOs, will be necessary to understand the HERC1-specific function in these cells.

Studies carried out with male and female animals allow sex-based analysis of bone homeostasis. Our study confirms previous observations showing a greater decrease in distal femoral trabecular volume with age in female mice than in male mice [53]. As a novelty, our study reports a greater sensitivity to developing osteopenia in young *Herc1*-KO female mice than in WT controls. Sex hormone analysis showed a significant decrease in testosterone and dihydrotestosterone levels in these animals, resulting in an inverse correlation between osteopenia and androgens levels. Previous studies have shown that androgens suppress RANKL-induced osteoclast formation [54]. Orchiectomy and androgen receptor deletion causes bone loss [55]. Thus, androgen deficiency leads to an increase in osteoclastic bone resorption and a progressive decrease in bone mineral density. Given the association between androgen deficiency and decreased trabecular bone mass, androgens may exert a protective effect on trabecular bone [55]. Future studies should delve into this hypothesis and into the molecular mechanisms associated with androgen decline in young female KO mice.

Osteocytes are the most abundant cells in bone and are the major orchestrators of bone remodeling and mineral homeostasis [56]. They are also the main source of FGF23, which binds to its receptor KLOTHO-FGFR1c in its target organs. In the kidneys, elevated FGF23 levels inhibit phosphate transporters, causing phosphaturia and hypophosphatemia [57], as observed in patients with chronic kidney disease [58]. In the parathyroid gland, FGF23 inhibits gene expression of parathyroid hormone, which the body produces in response to low levels of serum calcium, acting on the bone and kidney to increase serum calcium levels. Several studies have demonstrated an association for higher serum FGF23 levels with left ventricular hypertrophy, impaired vasoreactivity, and increased arterial stiffness [59–61]. The increased expression of *Fgf23* mRNA in osteocytes derived from *Herc1*-KO mice suggests an association between HERC1 loss-of-function and renal and cardiovascular disease.

Our findings may have important physiological and clinical implications. Physiologically, we identify HERC1 as a fine-tuning regulator of bone homeostasis. In a preclinical model, we also found that HERC1 loss-of-function caused osteopenia in adulthood. Therefore, HERC1 is a candidate to be evaluated to identify the etiology of osteopenia. In addition, our data could have implications for preventive medicine. Genetic analysis of HERC1 at an early age would help identify individuals with HERC1 loss of function and candidates for developing MDFPMR syndrome. In these cases, it would be interesting to analyze whether the expected bone alterations could be prevented or limited by the use of RANKL inhibitors. Compounds that inhibit RANKL-induced osteoclastogenesis, such as the RANKL-blocking monoclonal antibody Denosumab [62], could be therapeutic candidates to prevent or limit the expected bone alterations. Our data also point to the potential for specific inhibitors against ERK, p38, and RAF as therapeutic candidates.

In summary, this study identifies HERC1 as a regulator of bone remodeling, associates HERC1 deficiency with osteopenia, and suggests molecular targets for therapeutic strategies.

## Supplementary information

Supplementary figures

Original Data File

Reproducibility checklist

## Data Availability

The experimental data sets generated and/or analyzed during the current study are available from the corresponding author upon reasonable request. No applicable resources were generated during the current study.
