# Supplementary material for: HERC1 deficiency causes osteopenia through transcriptional program dysregulation during bone remodeling
Source: Cell Death Dis. 2023 Jan 12;14(1):17. doi: 10.1038/s41419-023-05549-x (PMC9837143; doi:10.1038/s41419-023-05549-x)
Supplement: Supplementary file 1 — Supplementary figures [file 41419_2023_5549_MOESM1_ESM.pdf]

A

Males

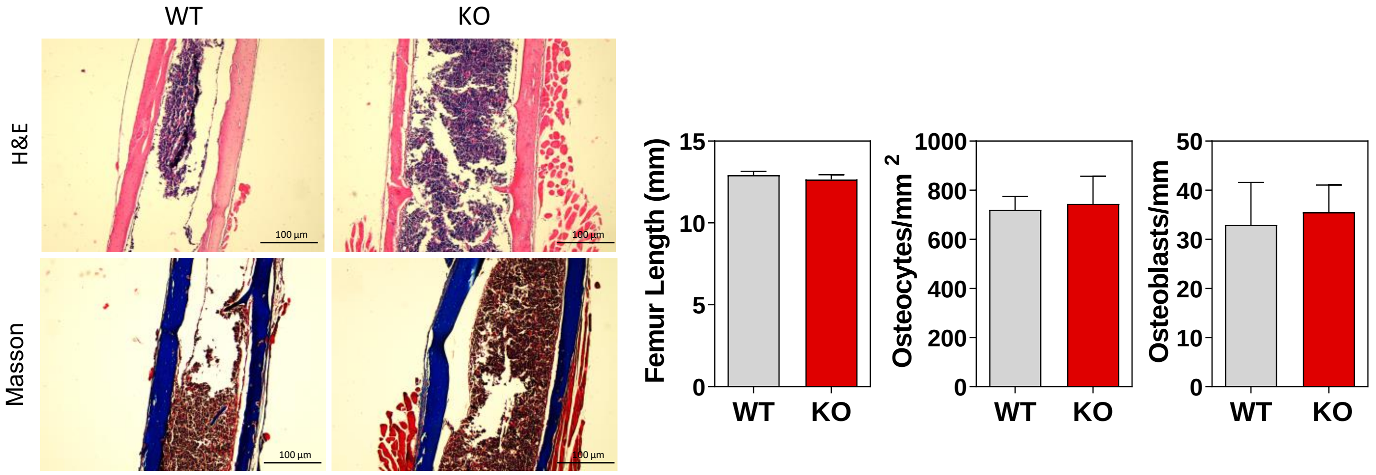

B

Females

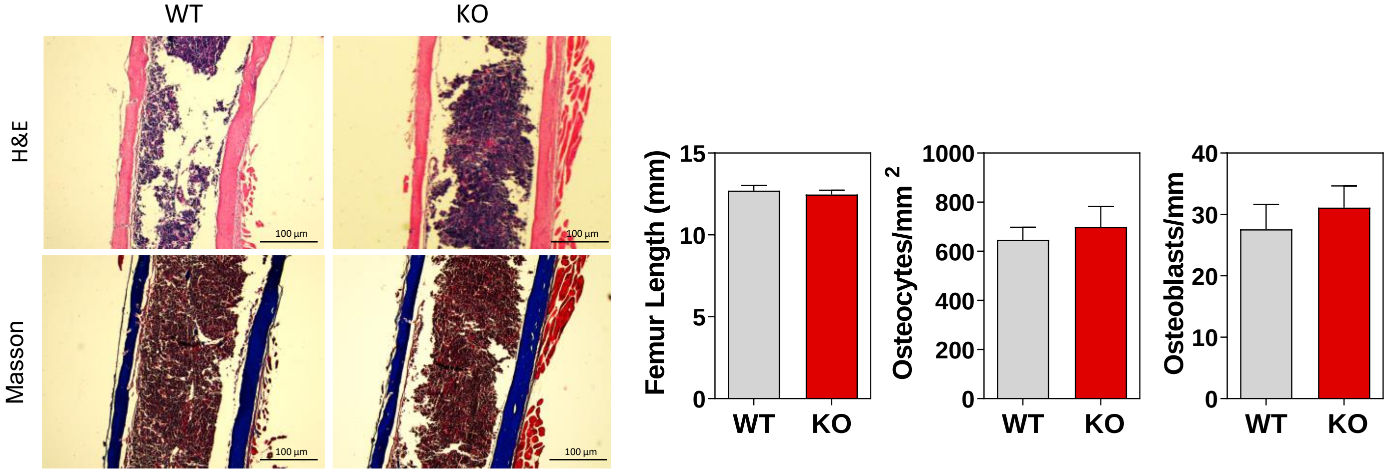

Femur sections from male **(A)** and female **(B)** 8 week-old mice stained with H&E or Masson Trichrome. In the right panels, femur length (n=5-8), osteocyte number per mm^2 of cortical bone (n=3-4) and osteoblast number per mm of trabeculae surface (n=4) of WT and KO mice, from both males **(A)** and females **(B)**.

Supplementary Figure 1

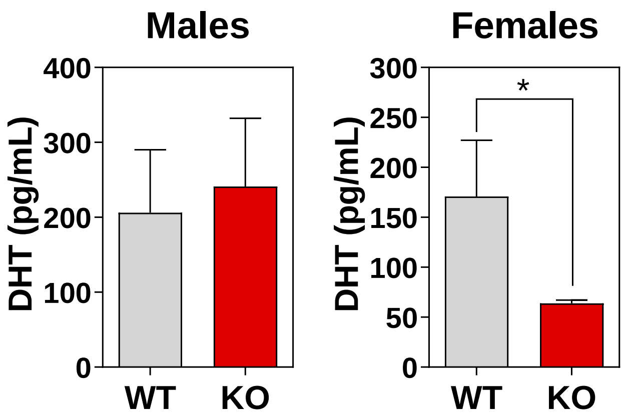

Dihydrotestosterone (DHT) levels in the serum of male and female WT and KO 8 week-old mice. DHT was measured by ELISA (n=7-12). Data are expressed as mean ± SEM. Significant differences are relative to WT. *$p<0.05$.

Supplementary Figure 2
