# Supplementary material for: HERC1 deficiency causes osteopenia through transcriptional program dysregulation during bone remodeling
Source: Cell Death Dis. 2023 Jan 12;14(1):17. doi: 10.1038/s41419-023-05549-x (PMC9837143; doi:10.1038/s41419-023-05549-x)
Supplement: Supplementary file 2 — Original Data File [file 41419_2023_5549_MOESM2_ESM.pdf]

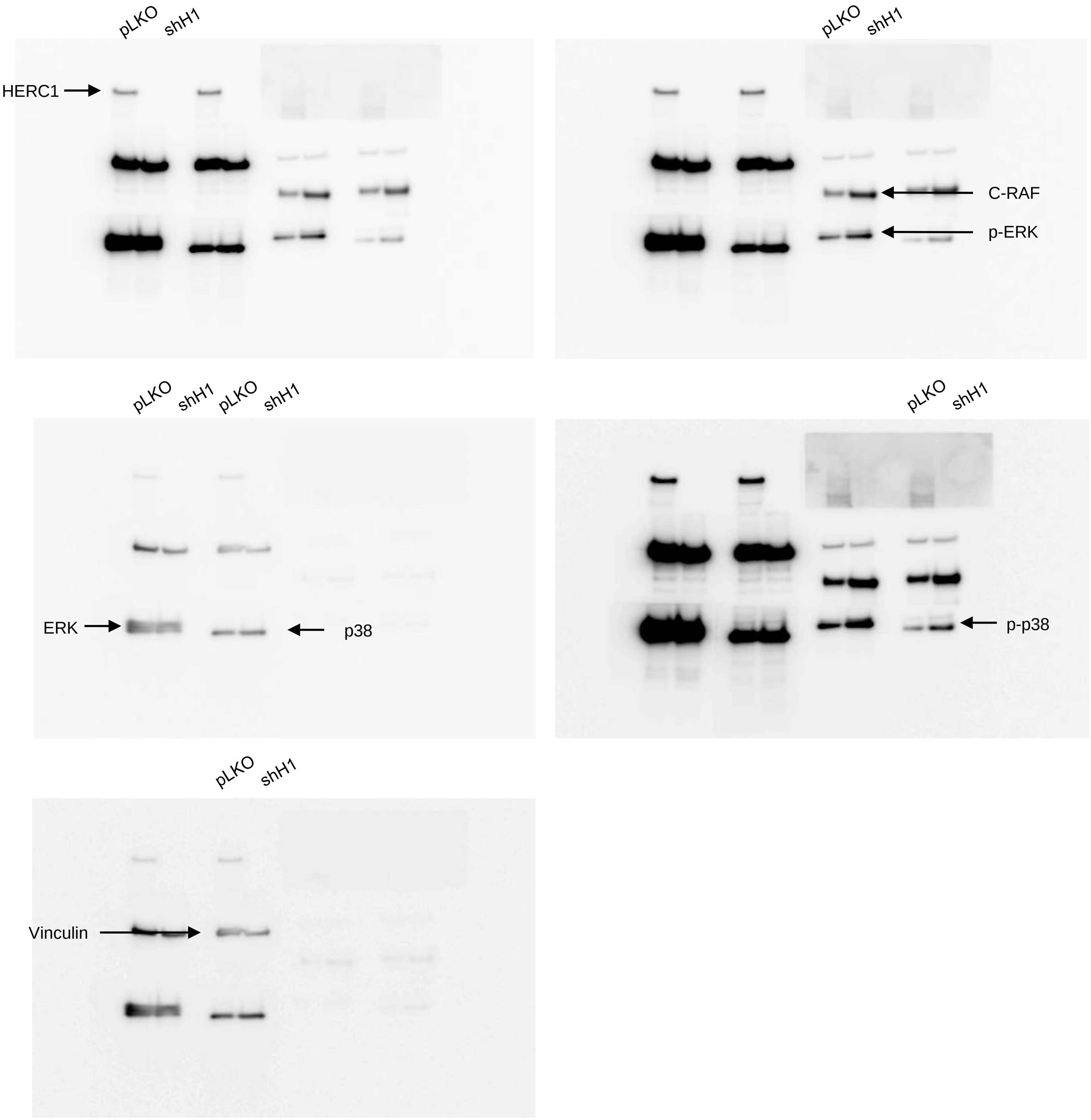

Uncropped Western Blot Figure 1A

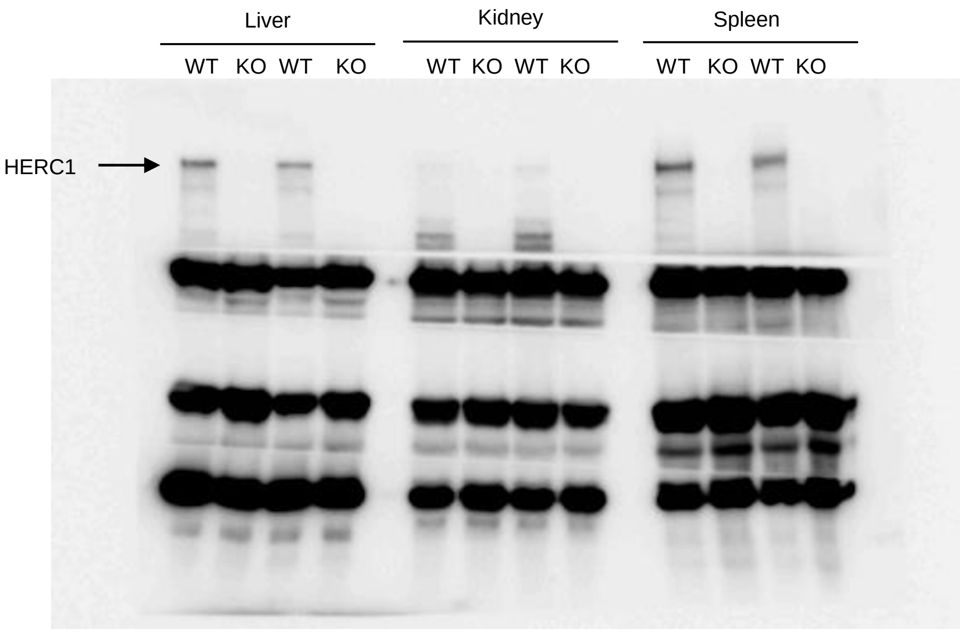

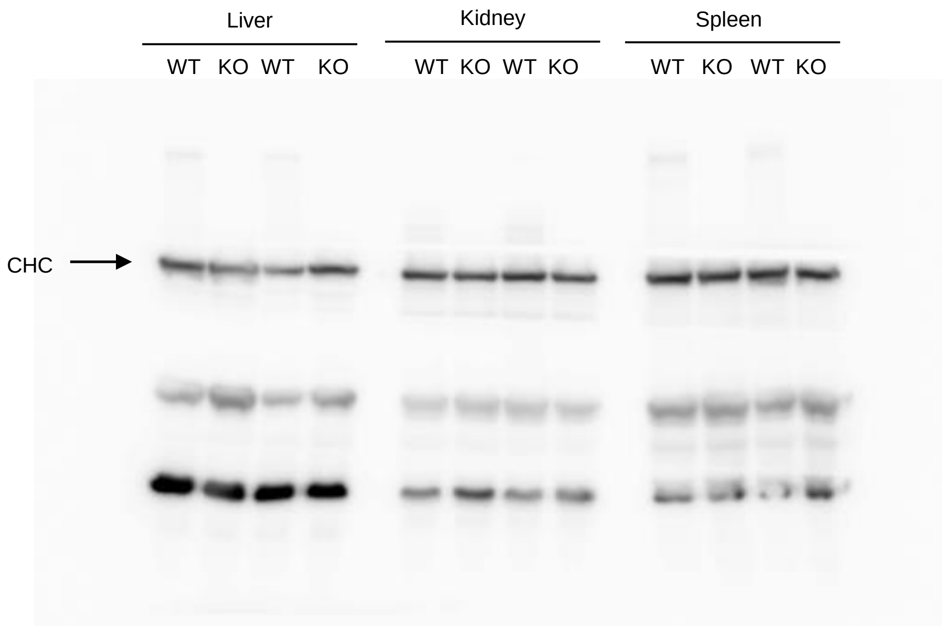

Uncropped Western Blot Figure 2C

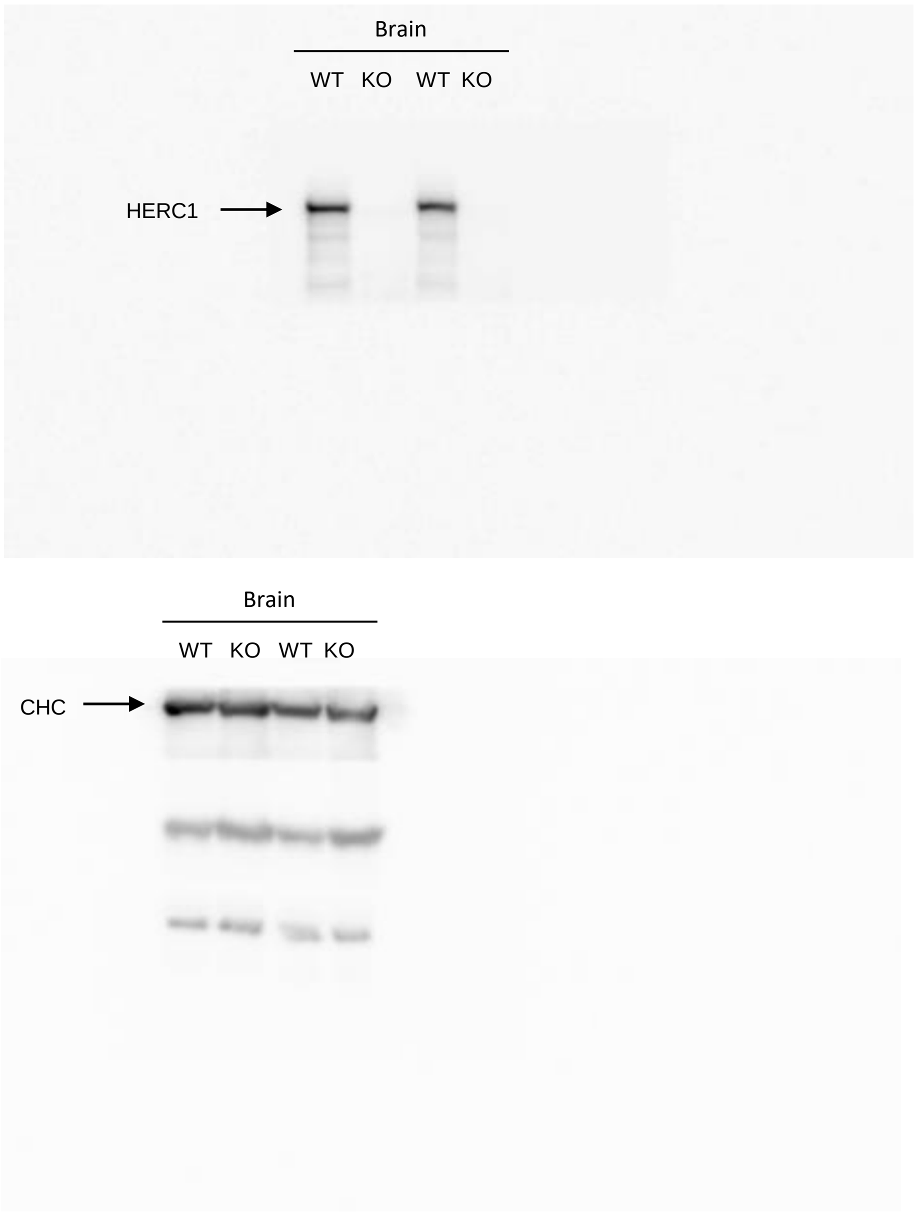

Uncropped Western Blot Figure 2C
